# A regional audit system for stillbirth: a way to better understand the phenomenon

**DOI:** 10.1186/s12884-019-2432-2

**Published:** 2019-08-05

**Authors:** Gaia Po’, Francesca Monari, Filippo Zanni, Giovanni Grandi, Camilla Lupi, Fabio Facchinetti, for the Stillbirth Emilia-Romagna Audit Group, Luciano Mancini, Laura Lugli, Chiara Lanzoni, Laura Sgarbi, Claudio Chiossi, Federica Ricchieri, Capucci Roberta, Raffaella Contiero, Gianpaolo Garani, Massimo Pedriali, Sonia Rossi, Sergio Fini, Massimo Di Bartolo, Daniele Radi, Alessandra Vancini, Anna Donati, Eleonora Guadalupi, Francesca Righetti, Angela Salerno, Guido Cocchi, Raffaella Morandi, Liliana Gabrielli, Claudio Graziano, Marco Seri, Giacomo Caprara, Salfi Nunzio Cosimo Mario, Francesca Fantuz, Federica Ferlini, Elisa Righi, Deborah Silvestrini, Franco Foschi, Stefania Fieni, Tiziana Frusca, Alice Ferretti, Letizia Galli, Cinzia Magnani, Enrico Silini, Letizia Balduzzi, Melissa Bellini, Anna Maria Rodolfi, Maria Paola Sgarabotto, Giorgia Fragni, Giuseppina Comitini, Maria Paola Bonasoni, Loredana Fioroni, Cristina Rozzi, Antonella Tuzio, Ida Vito, Palma Mammoliti, Elena De Ambrosi, Monica Ricci, Angela Bandini, Chiara Belosi, Claudia Muratori, Silvia Zago, Alessandra Turci, Marisa Vitarelli

**Affiliations:** 1 Obstetrics and Gynecology Unit, Mother-Infant and Adult Department of Medical and Surgical Sciences, University of Modena and Reggio Emilia, Via del Pozzo 71, 41125 Modena, Italy; 2 Health facilities, Technologies and Information Systems Unit, Emilia-Romagna Region, Viale Aldo Moro 21, 40127 Bologna, Italy

**Keywords:** Stillbirth, Perinatal audit, Quality of care, Causes of death

## Abstract

**Background:**

Implementation of high-quality national audits for perinatal mortality are needed to improve the registration of all perinatal deaths and the identification of the causes of death. This study aims to evaluate the implementation of a Regional Audit System for Stillbirth in Emilia-Romagna Region, Italy.

**Methods:**

For each stillbirth (≥ 22 weeks of gestation, ≥ 500 g) occurred between January 1, 2014 to December 1, 2016 (*n* = 332), the same diagnostic workup was performed and a clinical record with data about mother and stillborn was completed. Every case was discussed in a multidisciplinary local audit to assess both the cause of death (ReCoDe classification) and the quality of care. Data were reviewed by the Regional Audit Group. Stillbirth rates, causes of death and the quality of care were established for each case.

**Results:**

Total stillbirth rate was 3.09 per 1000 births (332/107,528). Late stillbirth rate was 2.3 per 1000 (251/107,087). Sixteen stillbirths were not registered by the Regional Birth Register. The most prevalent cause of death was placental disorder (33.3%), followed by fetal (17.6%), cord (14.2%) and maternal disorders (7.6%). Unexplained cases were 14%. Compared to local audits, the regional group attributed different causes of death in 17% of cases. At multivariate analysis, infections were associated with early stillbirths (OR 3.38, CI95% 1.62–7.03) and intrapartum cases (OR 6.64, CI95% 2.61–17.02). Placental disorders were related to growth restriction (OR 1.89, CI95% 1.06–3.36) and were more frequent before term (OR 1.86, CI95% 1.11–3.15). Stillbirths judged possibly/probably preventable with a different management (10.9%) occurred more frequently in non-Italian women and were mainly related to maternal disorders (OR 6.64, CI95% 2.61–17.02).

**Conclusions:**

Regional Audit System for Stillbirth improves the registration of stillbirth and allows to define the causes of death. Moreover, sub-optimal care was recognized, allowing to identify populations which could benefit from preventive measures.

## Background

Despite the large number of stillbirths (SB) occurring every year [1, 2], global attention regarding this issue is still insufficient. SB rates have declined more slowly since 2000 than both maternal mortality and mortality in children younger than 5 years [3]. Evidence suggests that this gap can be narrowed by improving SB registrations, data collection, investigation into the cause of death, reducing social disadvantages and preventing modified risk factors [4]. The Every Newborn Action Plan (ENAP), endorsed by the United Nations, aims to reduce the SB rate to 12 or fewer per 1000 births in every country by 2030 [5]. Moreover, ENAP suggests all countries to implement high quality national audits for perinatal mortality, which translates into improvements in quality of care, and the registration of all perinatal deaths together with the identification of the cause of death. Achieving these goals requires optimal diagnostic testing and multidisciplinary review as part of a high-quality perinatal mortality audit. According to a recent report [6], countries that have implemented national perinatal audit programs have achieved a reduction in SB rates. In addition, they have found an unacceptably high proportion of cases with elements of sub-optimal care. Despite the benefits of perinatal audit, still today few Nations have implemented this scheme [7–9].

The existence of a large number of classification systems does not always lead to the identification of the cause of death, leaving many cases unexplained [10]. Establishing an accurate cause of death is necessary for parents to understand why their baby died, help them to cope with the death and reduce the risk of recurrence [11, 12].

A national perinatal audit program is currently lacking in Italy. In Emilia-Romagna (ER), a region in the North of the country, a Regional SB Audit System, managed by a multidisciplinary panel, has been implemented since 2014 when the Regional Council approved a resolution [13] to set up multidisciplinary working groups, to perform local and regional perinatal audits.

This study describes the process of the SB audit programme and presents the results after the first 3 years of implementation.

## Methods

### The process of audit

This audit project started after preparatory steps, established between 2012 and 2014. An “ad hoc” Commission designed the SB clinical record and the complete diagnostic work-up for their use in every Obstetrics Units. Then, a 2-h e-learning course was offered to each professional (obstetricians, neonatologists and midwives) in every hospital to teach how to complete stillborn records and how to carry out diagnostic protocol. From 2014 to 2016 each case of SB underwent this type of evaluation in all 29 hospitals. The investigation is still ongoing.

The diagnosis of SB was based on the World Health Organization (WHO) recommendation [14] and was defined as fetal death at 22 weeks (154 days) of gestation or greater, or birthweight of 500 g if the gestational age was unknown. According to WHO’s recommendation, late SB was defined as a fetus of 1000 g and/or 28 weeks of gestation or greater and early SB as a fetus with a gestational age between 22 and 27.

Maternal information (demographics, obstetric history, presence of risk factors, antenatal investigations, such as the number of medical examinations and ultrasounds) were collected. Date and gestational age at delivery, birthweight, placenta weight, circumstances of the SB, neonate external inspection carried out by a neonatologist, were recorded together with the list of tests done after the diagnosis of SB. Clinical records with data about mother and stillborn were completed by the physician attending the women.

Diagnostic work-up included placental histology, stillborn autopsy, microbiological evaluation (vaginal, placental, fetal oropharyngeal swab/blood culture), maternal blood tests, maternal serologic status for infections, cytogenetic analysis (karyotype and, only in specific case, CGH-array), flow cytometry for the research of fetal-maternal haemorrhage and neonate inspection by a neonatologist.

Primary and associated relevant conditions at death were categorized using ReCoDe classification [15]. This system is based on 9 groups (fetus, cord, placenta, amniotic fluid, uterus, mother, intrapartum, trauma, unclassified). This classification was developed to better understand the clinically relevant conditions for SB regardless of whether an underlying cause was established. A comparison of different classifications demonstrated that ReCoDe performs better in terms of retaining important information and ease of use, reporting also a low proportion of unexplained cases [16].

Quality of care evaluation was also discussed according to Confidential Enquiry into Stillbirths and Deaths in Infancy (CESDI) grade [17]: (0: no substandard care; 1: substandard care, different management would have made no difference to outcome; 2: substandard care, different management might have made a difference to outcome; 3: substandard care, different management would have reasonably been expected to have made a difference to outcome). A death is considered potentially avoidable if the absence of the contributory factors may have prevented it. Relevant Italian Guidelines are used to evaluate the quality of care provided in relation to antenatal and intrapartum care.

Six local area audits, covering the entire regional territory, were organized twice a year to collect and discuss cases. The multidisciplinary team included at least an obstetrician, a neonatologist and a pathologist. Each team was led by a local coordinator, who recorded information on the results of investigations and compiled the cause of death after the local audit. He also checked every record and was responsible for their final compilation.

Local coordinators together with other specialists, such as microbiologists and geneticists, met every six months as the central multidisciplinary audit group. This group checked the number of SB comparing it to those recorded in Birth certificates, registered the causes of death and discussed cases defined as doubtful at the local audit. In the case of incomplete information, local coordinators were asked to do further investigations in order to complete the database. Data on the result of regional audit were managed and elaborated by the central coordinator.

The audit was paper-based. This assure the validity of the information collected which can be easily checked for each case by the local coordinators.

The present analysis of data was performed in agreement with the Regional Council’s resolution [13] and requested by the Birth Regional Commission in order to evaluate perinatal care in the Region. A preliminary analysis was published in the annual report on pregnancy care in Emilia-Romagna [18]. The study was approved by the Institutional Review Board. Informed consent for diagnostic work-up was not required because in Italy diagnostic investigation is mandatory by law in case of stillbirth (D.M. 7/2014 and D.P.C. 170/99). Patient and fetus privacy was ensured during the phase of data collection and analysis.

### Definitions and statistical analysis

Fetal growth restriction (FGR) was defined as a birth-weight below the 10th centile according to Italian Neonatal Study (INeS) chart [19] to categorize relevant conditions at death and below the 5th centile for multivariate analysis. Large for gestational age (LGA) was defined as a birth-weight over the 95th centile.

Gestational age was estimated based on the last menstrual period or on the first ultrasound examination, if the last menstrual period was unknown or unreliable.

Placental insufficiency was defined as the presence of histological features of functional impairment, such as placental hypoplasia, infarcts covering at least 10% of the placenta, diffuse villous hypoplasia, accelerated villous maturation, fetal vascular malperfusion and high grade chronic villitis.

Placental abruptio was defined as cause of death if there was clinical evidence of this condition and a histopathologic finding of retroplacental hematoma.

The death was attributed to infection if there was evidence of fetal infection, e.g. pathogen isolation in blood culture/oropharyngeal swab and/or histological feature of fetal inflammatory response.

Chorioamnionitis was defined as a cause of death if associated with funisitis at histological examination and/or associated with clinical signs.

Maternal diabetes was the cause of death based on histopathological signs of impaired glucose metabolism and clinical signs of poor controlled maternal diabetes (e.g. macrosomia, polidramnios, elevated blood sugar despite the therapy, high glycate haemoglobin three months after death). In the same way, hypertensive disorders were defined as cause of death based on severity, poor/absent clinical control and histopathological signs of hypertension. Otherwise diabetes and hypertensive disorders were simply recorded as associated conditions.

Cord constricting loop or knots were considered the cause of death in those cases with histopathological finding supporting this cause, such as thrombosis immediately before and after the knot, or along the constricting loop.

A pre-pregnancy body mass index (BMI) ≥30 kg/m^2^ was defined as obesity and ≥ 25 kg/m^2^ as overweight.

Information was collected in a database. Because of privacy restrictions and to create a safe and secure environment for audit participants, the database was anonymous.

In order to calculate risk factors, data of SB were compared with livebirth data in the same period, using the Regional Birth Register, which simultaneously collects information about livebirths and stillbirths [18, 20, 21].

Data were analysed using statistical package StatView (v 5.01.98; SAS Istitute Inc., Cary, NC). Categorical variables are expressed as frequencies and percentages. Odd Risk (OR) with 95% confidence interval (CI) was computed when appropriate. A *p* value of 0.05 or less was considered significant. Multivariate analysis was performed in order to verify associations between condition of death and gestational age, birthweight and maternal/fetal disorders. They were adjusted for known risk factors for adverse pregnancy outcomes such as BMI, smoking, education level and maternal country of birth.

## Results

From 2014 to 2016, 332 SB occurred out of a total of 107,528 births, with a SB rate of 3.1 per 1000. Of these SB, 81 (24.4%) occurred before 28 weeks and 251 thereafter (75.6%), yielding a late SB rate of 2.3 per 1000. The SB rate constantly declined between 2014 (3.2 per 1000) and 2016 (3.0 per 1000). Seventeen cases (5.1%) occurred after the onset of labour and were considered intrapartum. Double-check led to the identification of 21 early SB which had not been recorded in the current Birth Register.

Fifteen cases (4.5%) originated from multiple pregnancies. Our population was heterogeneous in term of ethnicity: there were 190 (57.6%) Italian women, the others were from North Africa (11.5%), East Europe (10.3%), Sub-Saharan Africa (8.2%), Indian Subcontinent (7.6%) and other countries (4.8%).

Complete diagnostic protocol was applied in the majority of cases. Placental examination and autopsy were performed in 298 (90.3%) and 290 cases (87.8%), respectively.

Clinical records were available for all cases but two, seized by the judicial authority. For this reason, analyses were conducted on a population of 330 SB.

Risk factors for SB are reported in Table 1. After 41 weeks, the risk of fetal demise was lower than in preterm and full-term pregnancies (OR 0.3, CI95% 0.1–0.8). A progressively increasing risk was identified in overweight (OR 1.42, CI95% 1.07–1.86) and obese women (OR 1.96, CI95% 1.40–2.74). Women from Indian Subcontinent, North and Sub-Saharan Africa presented a higher risk of SB than Caucasian and above all Sub-Saharan Africa women had an almost tripled risk (OR 2.9, CI95% 1.94–4.35). Finally, women who had a previous SB carried a greater risk of recurrence (OR 2.62, CI95% 1.34–5.14). The risk was not significantly increased for smoking in pregnancy, multiple gestations, maternal age and education.

**Table 1:** Risk factors for stillbirth compared to live births

Risk factors	Live births^a^ (number)	Stillbirths^a^ (number)	Rate (‰)	OR	CI95%
Gestational age category (weeks)					
39–40	55,975	54	1.0	reference	
≥ 41	17,262	5	0.3	0.3	0.1–0.8
Pregestational Maternal BMI (kg/m^2^)					
18–24	67,268	171	2.5	reference	
25–29	19,011	68	3.6	1.42	1.07–1.86
≥ 30	8620	43	5.0	1.96	1.40–2.74
Maternal nationality					
Italy	73,854	190	2.6	reference	
East Europe	13,820	34	2.5	0.96	0.66–1.38
North Africa	7690	38	4.9	1.6	1.35–2.72
Indian Subcontinent	3752	25	6.6	2.6	1.70–3.94
Sub-Saharan Africa	3616	27	7.4	2.9	1.94–4.35
Stillbirth recurrence					
No previous stillbirth	48,574	194	4.0	reference	
Previous stillbirth	859	9	10.4	2.62	1.34–5.14

^a^ data from Regional Birth Register

Table 2 shows the distribution of causes of death in our cohort. The Regional Audit Program reviewed all cases previously evaluated by local groups and established a different cause of death in 54 (16.4%) cases. Disagreement on the causes of death between local and central committees were more frequent during the first year of the project and progressively reduced in the following years, when local committees improved their ability in interpreting the results of the investigations and classifying the cause of deaths, thanks to discussions during the meetings (data not shown). Placental disorders were the most frequent relevant condition (33.3%), followed by the fetus (17.6%), cord (14.2%) and maternal disorders (7.6%). Forty-seven (14.2%) cases remained unexplained even though the diagnostic protocol had been carried out. However, it was not possible to assign a cause of death in 14 cases (4.2%) because the diagnostic work-up was incomplete.

**Table 2:** Distribution of causes of death according to ReCoDe classification

Relevant condition at death			N	%	%
Group A: Fetus	Lethal congenital anomaly		16	4.8	17.6
	Infection		20	6.1	
	Non immune hydrops		1	0.3	
	Isoimmunisation		2	0.6	
	Fetomaternal haemorrhage		6	1.8	
	Twin-twin transfusion		2	0.6	
	Fetal growth restriction		11	3.3	
Group B: Cord	Constricting loop or knot		20	6.1	14.2
	Velamentous insertion		1	0.3	
	Cord: other	Funisitis	2	0.6	
		Iperspiralisation	3	0.9	
		Stenosis	4	1.2	
		Thrombosis	14	4.2	
		Other	3	0.9	
Group C: Placenta	Abruptio		47	14.2	33.3
	Placental insufficiency		56	17.0	
	Placenta: other		7	2.1	
Gruppo D: Amniotic fluid	Chorioamnionitis		19	5.8	5.8
Group E: uterus	Rupture		6	1.8	1.8
Group F: Mother	Diabetes		13	3.9	7.6
	Essential hypertension		2	0.6	
	Hypertensive disorders in pregnancy		9	2.7	
	Lupus or antiphospholipid syndrome		1	0.3	
Group G: Intrapartum	Asphyxia		3	0.9	0.9
Group H: Trauma	External		1	0.3	0.3
Group I: Unclassified	No relevant condition identified		47	14.2	18.5
	No information available		14	4.2	
TOTAL			330	100.0	100.0

Associated relevant conditions at death were found in 329 cases and the most frequent one was FGR which was ascertained in 49 cases (14.9%).

For multivariate analysis, relevant conditions at death have been categorized in seven groups: placental disorders (110 cases), unexplained (61 cases), cord accidents (45 cases), infections (including proven fetal infection, histologic funisitis and chorioamnionitis −39 cases-), fetal disorders (38 cases), maternal disorders (25 cases) and others (12 cases).

Infections were associated with early intrauterine fetal death (OR 3.38, CI95% 1.62–7.03) while placental disorders were related to preterm SB (OR 1.86, CI95% 1.11–3.15) and FGR (OR 1.89, CI95% 1.06–3.36). Furthermore, maternal disorders were associated with overweight (OR 3.38, CI95% 1.33–8.6) and LGA (OR 4.26, CI95% 1.07–12.87).

No significant association was found between causes of death and smoking (although abruptio was more frequent in this group), level of education, multiple pregnancies and maternal country of birth, even though women from Indian Subcontinent had a high proportion of SB due to placental disease (48%).

The quality of care provided during pregnancy and labour has been assessed as shown in Table 3. Elements of substandard care were present in 48 (14.5%) cases. A different management might have made or would have reasonably made a difference to outcome in 36 (10.9%) cases. Elements of sub-optimal care were identified only in antepartum cases. The first cause of death among such cases was placental insufficiency (30.5%), followed by maternal disorders -diabetes, hypertension disorders, antiphospholipid syndrome- (25%). At multivariate analysis the group of maternal disorders was associated with substandard care of grade 2 and 3 (OR 6.64, CI95% 2.61–17.02). No significant association has been found for the remaining causes of death. Eleven women (30.5%) had a delayed access to antenatal care or missed the antenatal appointments because ignored their pregnancy or either refused hospitalisation. In 9 cases (25%) clinicians failed to diagnose or manage diabetes or hypertensive disorders. Six cases (16.6%) affected by fetal growth restriction were either not detected or managed inappropriately. In 9 cases different suboptimal care factors were identified, e.g. entering the pregnancy with an elevated BMI or continuing smoking during pregnancy.

**Table 3:** Quality of care during pregnancy and labour according to CESDI grade

Grade		Number (%)
Grade 0	no substandard care	260 (78.8)
Grade 1	substandard care, different management would have made no difference to outcome	12 (3.6)
Grade 2	substandard care, different management might have made a difference to outcome	27 (8.2)
Grade 3	substandard care, different management would have reasonably been expected to have made a difference to outcome	9 (2.7)
	data not available	22 (6.7)
Total		330 (100)

**Fig. 1:**
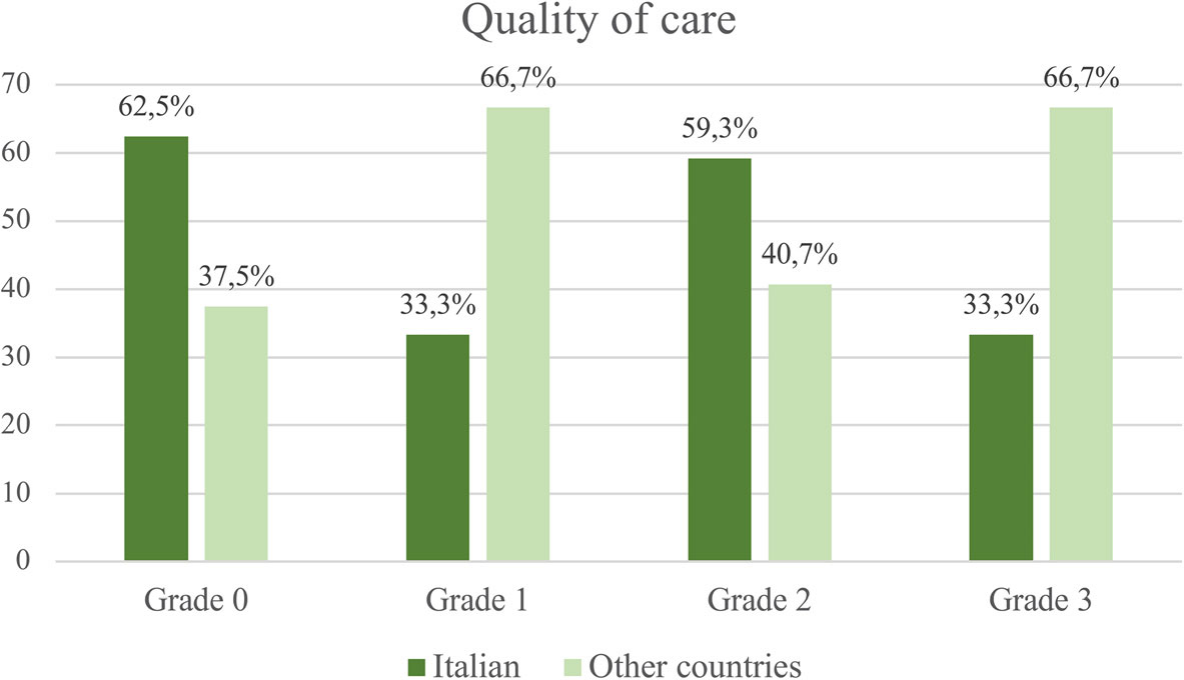
Distribution of women’s country of birth in relation to quality of care

Among the 9 cases evaluated of grade 3, seven occurred in racial minorities and only 2 in Italian women. Figure 1 shows the distribution of women’s country of birth in relation to quality of care grading. The majority of cases without substandard care occurred in Italian women, while most preventable stillbirths (grade 3) occurred in migrant women born in other countries.

The leading cause of death in intrapartum SB was infection (41.1%), followed by asphyxia (11.8%), uterine rupture (11.8%) and others (17.7%). Unexplained cases were 17.6%. Among the seven intrauterine fetal deaths caused by infection, 5 occurred before 28 weeks. At logistic regression, intrapartum SB were associated with infections (OR 7.15, CI95% 2.35–21.77), while there was no association with inadequate care (grade 2 and 3).

## Discussion

The audit process allowed a more realistic evaluation of stillbirth phenomenon, highlighting the underestimation of early SB, due to the present Italian law which defines miscarriage as products of conception prior to 180 days of development (25 weeks and 5 days). Thus, we explained the discrepancy of 21 SB recorded in the audit process, and not in the Birth Register, with the fact that stillbirths occurred between 22 weeks and 25 weeks and 5 days were registered as miscarriage, according to the Italian law, instead of using WHO’s definition for stillbirths.

Late SB rate in ER was one of the lowest rates in Europe after Iceland, Denmark, Finland, the Netherlands, Croatia, Norway and Portugal [4].

Most of the risk factors for SB known in literature are confirmed by our study - overweight/obesity [22], racial disparity [2, 4] and previous SB - [23–25]. On the other hand, while post-term pregnancy is widely recognized as a condition of higher risk for SB [26], this is not confirmed in our population where the risk decreased after 41 weeks compared to 39–40 weeks and no fetal death occurred after 42 weeks. We hypothesized that such finding could be related to the strict surveillance of pregnancies above term in our Region. Indeed, there is a weekly monitoring and scheduled induction for pregnancies with medical indications, like gestational diabetes, hypertensive disorders and late term pregnancy (> 41 weeks) [27–29].

Placental disorders were the leading cause of death in our cohort and explained one-third of stillbirths in line with other authors which reported placental dysfunction as a major contributor [30–32]. Moreover, fetal disorders were responsible for about one case out of six and FGR was the principal relevant condition at death in only 3% of cases. Such frequency is much lower than the one reported by Vergani et al. (17%) [16] with the same classification system. When we attributed the relevant condition at death, we considered placental disorders as a logic antecedent of FGR and then SB, believing that growth restriction could be the cause of death only in the absence of other conditions. Indeed, considering also the associated factors, the whole rate of FGR (14.9%) was similar to the one in Vergani’s group. Our results are in general agreement with those of Ego et al. [33] who previously adopted the same logic to classify cause of stillbirths with ReCoDe, i.e. the incidence of placental causes doubled becoming almost 25% when FGR was considered as a cause only in the absence of other conditions.

Infections are another significant cause of death, counting for almost 12%. This is particularly true for early SB as previously reported by other authors [34, 35].

On the other hand, the Audit System could not explain a significant number of cases (about one case out of seven). This rate was close to those previously reported by English [15], Italian [16] and Dutch [31] studies. Therefore, despite the existence of different classification systems [10], a consistent number of SB remains unexplained.

It is important to underline that the diagnostic workup was performed in every hospital, limiting unexplained stillbirths due to lack of investigation to < 5%. At the same time, placental pathology and fetal autopsy were extensively carried out.

Based on current guidelines, substandard care factors were identified by the multidisciplinary panel in a significant number of cases where a different management might have made a difference to outcome or would have reasonably been expected to have made a difference to outcome. In high-income countries, few perinatal audits evaluated the quality of care in relation to SB. A national audit in the Netherlands identified such inappropriateness in 8% of more than 700 term perinatal deaths [9]. Also a review in New Zealand identified that up to 15% of deaths are potentially avoidable [8]. The Euronatal study [36], which included quality of care evaluation in 10 European regions between 1993 and 1998, found an even higher percentage of sub-standard care factors than previously reported. It seems likely that the above differences could be partly explained by quality of care improvement during the past 20 years.

In our study, the main issues recognised as areas for improvement in antenatal care provision included the access to antenatal care for disadvantages minorities and the identification and management of maternal disorders. Indeed, almost all cases with quality of care graded 3 occurred in women with poor access to antenatal care, also because of unintended pregnancies. This confirms that negative outcome did not always depend on care offered but pertained to very disadvantaged minorities. Therefore, it is mandatory to increase early access to the antenatal care system in the effort to minimize racial and ethnic disparities.

Furthermore, the association between the presence of maternal disorders and inadequate care should stimulate actions looking at the improvement of the management of high-risk pregnancies. Improving detection and management of metabolic and hypertensive disorders, seems of paramount importance also in reviewing the correlation between SB-related maternal disorders and overweight/obesity.

As expected in western countries [2], intrapartum cases were limited to 5% and were associated with appropriate care in the vast majority of the cases. At logistic regression, such deaths were associated with infections, namely before 28 weeks, as also reported by others [32]. Indeed, it is possible that infections may have induced preterm labour and determined fetal death because of extremely premature labour.

A limitation of the audit system is that it is time consuming. Another one is that despite the presence of multidisciplinary professionals, there is no external validation. Furthermore, histological exams were performed locally by different pathologists whose expertise in perinatal pathology was heterogenous. The importance of placental pathology, confirmed also by our work, supports the importance of having pathologists trained and specialized in perinatal pathology. Moreover, deaths occurring in the first neonatal week were not included. Finally, we do not have sufficient data to assess the impact of the validity of each investigation.

This study has several strengths. Comparison between cases detected by the audit project and current Birth Register led to a precise counting of each and every SB. Moreover, a homogeneous work-up was performed and information was prospectively collected, ensuring a high quality of data which is difficult to reach in large database. Moreover, causes of death were assigned by consensus among multidisciplinary panel of experts in maternal-fetal medicine and this guaranteed a high accuracy results.

## Conclusions

These data demonstrate that it is possible to implement a Regional Audit System of Stillbirth as recommended by international institutions [37]. Overall results are useful to understand local reality, in order to plan interventions towards specific populations.

## Data Availability

The dataset used and/or analyzed during the current study is available from the corresponding author on reasonable request.

## Abbreviations

ER: Emilia Romagna
FGR: Fetal growth restriction
LGA: Large for gestational age
SB: Stillbirths
WHO: World Health Organization

## References

[1] Blencowe H, Cousens S, Jassir FB (2016). National, regional, and worldwide estimates of stillbirth rates in 2015, with trends from 2000: a systematic analysis. Lancet Glob Health.

[2] Lawn JE, Blencowe H, Waiswa P (2016). For *The Lancet* ending preventable stillbirths series study group with *The Lancet* stillbirth epidemiology investigator group. Stillbirths: rates, risk factors, and acceleration towards 2030. Lancet.

[3] You D, Hug L, Ejdemyr S (2015). Global, regional, and national levels and trends in under-5 mortality between 1990 and 2015, with scenario-based projections to 2030: a systematic analysis by the UN inter-agency Group for Child Mortality Estimation. Lancet.

[4] Flenady V, Wojcieszek AM, Middleton P (2016). For the lancet ending preventable stillbirths study group and the lancet stillbirths in high-income countries Investigator Group. Stillbirths: recall to action in high-income countries. Lancet.

[5] World Health Organization (2014). Every newborn: an action plan to end preventable deaths.

[6] Norris T, Manktelow BN, Smith LK, Draper ES (2017). Causes and temporal changes in nationally collected stillbirth audit data in high-resource settings. Semin Fetal Neonatal Med.

[7] Draper ES, Kurinczuk JJ, Kenyon S, editors. Term, singleton, normally formed, antepartum stillbirth. Leicester: infant mortality and morbidity studies, department of health sciences. University of Leicester; 2015.

[8] Perinatal and Maternal Mortality Review Committee (2015). Ninth annual report of the perinatal and maternal mortality review committee: reporting mortality 2013.

[9] Eskes M, Waelput AJM, Erwich JJHM (2014). Term perinatal mortality audit in the Netherlands 2010-2012: a population-based cohort study. BMJ Open.

[10] Leisher SH, Teoh Z, Reinebrant H (2016). Seeking order amidst chaos: a systematic review of classification systems for causes of stillbirth and neonatal death, 2009–2014. BMC Pregnancy Childbirth.

[11] Monari F, Pedrielli G, Vergani P (2016). Adverse perinatal outcome in subsequent pregnancy after stillbirth by placental vascular disorders. PLoS One.

[12] Meaney S, Everard CM, Gallagher S, O’Donoghue K (2017). Parents’ concerns about future pregnancy after stillbirth: a qualitative study. Health Expect.

[13] Emilia-Romagna R. Dgr 533/2008 “Percorso nascita: direttiva regionale alle Aziende sanitarie”. Full text. http://salute.regione.emilia-romagna.it/documentazione/leggi/regionali/dgr-2127-2016/dgr-533-2008-nascita/view.

[14] Zupan J, Åhman E (2006). Neonatal and perinatal mortality: country, regional and global estimates.

[15] Gardosi J, Kady SM, McGeown P, Francis A, Tonks A (2005). Classification of stillbirth by relevant condition at death (ReCoDe): population based cohort study. BMJ.

[16] Vergani P, Cozzolino S, Pozzi E (2008). Identifying the causes of stillbirth: a comparison of four classification systems. Am J Obstet Gynecol.

[17] Maternal and Child Health Research Consortium (2001). Confidential enquiry into stillbirths and deaths in infancy report. 8th annual (1998–1999).

[18] Lupi C, Perrone E, Basevi V (2017). La Nascita in Emilia-Romagna. 14° Rapporto sui dati del Certificato di Assistenza al Parto (CedAP) - Anno 2016.

[19] Bertino E, Spada E, Occhi L (2010). Neonatal anthropometric charts: the Italian neonatal study compared with other European studies. J Pediatr Gastroenterol Nutr.

[20] Basevi V, Battaglia S, Caranci N (2015). La Nascita in Emilia-Romagna. 12° Rapporto sui dati del Certificato di Assistenza al Parto (CedAP) - Anno 2014.

[21] Lupi C, Perrone E, Basevi V (2016). La Nascita in Emilia-Romagna. 13° Rapporto sui dati del Certificato di Assistenza al Parto (CedAP) - Anno 2015.

[22] Flenady V, Koopmans L, Middleton P (2011). Major risk factors for stillbirth in high-income countries: a systematic review and meta-analysis. Lancet.

[23] Samueloff A, Xenakis EM, Berkus MD, Huff RW, Langer O (1993). Recurrent stillbirth. Significance and characteristics. J Reprod Med.

[24] Sharma PP, Salihu HM, Kirby RS (2007). Stillbirth recurrence in a population of relatively low-risk mothers. Paediatr Perinat Epidemiol.

[25] Lamont K, Scott NW, Jones GT, Bhattacharya S (2015). Risk of recurrent stillbirth: systematic review and meta-analysis. BMJ.

[26] Rosenstein MG, Cheng YW, Snowden JM, Nicholson JM, Caughey AB (2012). Risk of stillbirth and infant death stratified by gestational age. Obstet Gynecol.

[27] ISS-SNLG, Istituto superiore di Sanità-Sistema linee guida. Gravidanza fisiologica. Linea guida. 2011. Roma: ISS. Full text: http://www.salute.gov.it/portale/documentazione/p6_2_2_1.jsp?lingua=italiano&id=1436

[28] Lenzi M. et al. Induzione al travaglio di parto. Revisione rapida e raccomandazioni. Bologna: Regione Emilia-Romagna, 2013. Full text:http://www.saperidoc.it/flex/cm/pages/ServeAttachment.php/L/IT/D/e%252F2%252F1%252FD.f2e8dc7d2d34be715517/P/BLOB%3AID%3D966/E/pdf.

[29] Paola Dallacasa et al. Ambulatorio gravidanza fisiologica a termine organizzato e gestito da ostetriche. Bologna: Regione Emilia Romagna, 2013. Full text: http://www.saperidoc.it/flex/cm/pages/ServeAttachment.php/L/IT/D/9%252F8%252Ff%252FD.182885b8e7c02a752fc6/P/BLOB%3AID%3D966/E/pdf.

[30] Varli IH, Petersson K, Bottinga R (2008). The Stockholm classification of stillbirth. Acta Obstet Gynecol Scand.

[31] Korteweg FJ, Erwich JJHM, Holm JP (2009). Diverse placental pathologies as the main causes of fetal death. Obstet Gynecol.

[32] Stillbirth Collaborative Research Network Writing Group (2011). Causes of death among stillbirths. JAMA..

[33] Ego A, Zeitlin J, Batailler P (2013). Stillbirth classification in population-based data and role of fetal growth restriction: the example of RECODE. BMC Pregnancy Childbirth.

[34] Goldenberg RL, McClure EM, Saleem S, Reddy UM (2010). Infection-related stillbirths. Lancet.

[35] Gibbs RS (2002). The origins of stillbirth: infectious diseases. Semin Perinatol.

[36] Richardus JH, Graafmans WC, Bergsjø P (2003). Suboptimal care and perinatal mortality in ten European regions: methodology and evaluation of an international audit. J Matern-Fetal Neonatal Med.

[37] World Health Organization (2016). Making every baby count: audit and review of stillbirths and neonatal deaths.

